# Contributions of h- and Na^+^/K^+^ Pump Currents to the Generation of Episodic and Continuous Rhythmic Activities

**DOI:** 10.3389/fncel.2021.715427

**Published:** 2022-02-04

**Authors:** Simon A. Sharples, Jessica Parker, Alex Vargas, Jonathan J. Milla-Cruz, Adam P. Lognon, Ning Cheng, Leanne Young, Anchita Shonak, Gennady S. Cymbalyuk, Patrick J. Whelan

**Affiliations:** ^1^School of Psychology and Neuroscience, University of St Andrews, St Andrews, United Kingdom; ^2^Hotchkiss Brain Institute, University of Calgary, Calgary, AB, Canada; ^3^Department of Neuroscience, University of Calgary, Calgary, AB, Canada; ^4^Neuroscience Institute, Georgia State University, Atlanta, GA, United States; ^5^Department of Comparative Biology and Experimental Medicine, University of Calgary, Calgary, AB, Canada; ^6^Department of Physics and Astronomy, Georgia State University, Atlanta, GA, United States

**Keywords:** episodic rhythms, central pattern generator, spinal cord, rhythmicity, dopamine, bursting, locomotor, elliptic episodic bursting

## Abstract

Developing spinal motor networks produce a diverse array of outputs, including episodic and continuous patterns of rhythmic activity. Variation in excitability state and neuromodulatory tone can facilitate transitions between episodic and continuous rhythms; however, the intrinsic mechanisms that govern these rhythms and their transitions are poorly understood. Here, we tested the capacity of a single central pattern generator (CPG) circuit with tunable properties to generate multiple outputs. To address this, we deployed a computational model composed of an inhibitory half-center oscillator (HCO). Following predictions of our computational model, we tested the contributions of key properties to the generation of an episodic rhythm produced by isolated spinal cords of the newborn mouse. The model recapitulates the diverse state-dependent rhythms evoked by dopamine. In the model, episodic bursting depended predominantly on the endogenous oscillatory properties of neurons, with Na^+^/K^+^ ATPase pump (*I*_Pump_) and hyperpolarization-activated currents (*I*_*h*_) playing key roles. Modulation of either *I*_Pump_ or *I*_*h*_ produced transitions between episodic and continuous rhythms and silence. As maximal activity of *I*_Pump_ decreased, the interepisode interval and period increased along with a reduction in episode duration. Decreasing maximal conductance of *I*_*h*_ decreased episode duration and increased interepisode interval. Pharmacological manipulations of *I*_*h*_ with ivabradine, and *I*_Pump_ with ouabain or monensin in isolated spinal cords produced findings consistent with the model. Our modeling and experimental results highlight key roles of *I*_*h*_ and *I*_Pump_ in producing episodic rhythms and provide insight into mechanisms that permit a single CPG to produce multiple patterns of rhythmicity.

## Introduction

Locomotor behaviors enable organisms to navigate and interact with their environment. These behaviors are remarkably diverse and flexible, but many species share a defined set of locomotor acts that are often engaged in specific contexts. For example, locomotor behavior during foraging is often episodic, interspersed with pauses to survey the environment. In contrast, pauses are less favorable during migratory locomotor behaviors, which are generally continuous to maximize distance traveled. Animals also need the capacity to rapidly switch between locomotor behaviors. When foraging animals sense a predatory threat, they need to respond quickly by initiating a freeze or escape response (Kim et al., 2017; Ferreira-Pinto et al., 2018). In mammals, the neural mechanisms that govern the generation of episodic and continuous locomotor bursts and transitions between them are unclear, but studies in fish and *Xenopus* tadpoles provide evidence that an interaction between descending commands, sensory modulation, and endocannabinoids contribute (Eaton et al., 2001; Sillar and Robertson, 2009; Berg et al., 2018).

Mounting evidence suggests that the spinal central pattern generator (CPG) encodes episodic and continuous locomotor patterns (Wiggin et al., 2012; Zhang and Sillar, 2012; Sharples and Whelan, 2017). Episodic and continuous bursting patterns of rhythmicity can be generated *in vitro* in neonatal mouse, zebrafish, and *Xenopus* tadpole spinal cord preparations by varying their neuromodulatory tone and excitability state (Wiggin et al., 2012; Zhang and Sillar, 2012; Sharples and Whelan, 2017). In larval zebrafish, features of episodic locomotor behaviors change during development from long, sporadic swimming episodes to shorter episodes with a more regular beat-and-glide pattern (Buss and Drapeau, 2001; Lambert et al., 2012). This switch, which coincides with the onset of foraging behaviors (Borla et al., 2002), is mediated by D_4_ dopamine receptors (Lambert et al., 2012), and patterns continue to mature through juvenile and adult stages of development (Müller et al., 2000; Gabriel et al., 2011). A similar developmental switch in locomotor behavior has been reported in *Xenopus* tadpoles (Currie et al., 2016). During early developmental stages, D_2_ dopamine receptors promote a sessile behavior, and at later stages, D_1_ receptors facilitate episodic swimming for filter-feeding (Clemens et al., 2012; Picton and Sillar, 2016). Swimming episodes in tadpoles have been linked to the activity-dependent Na^+^/K^+^ ATPase pump current (*I*_Pump_; Zhang and Sillar, 2012; Zhang et al., 2015), which interacts with hyperpolarization-activated cation current (*I*_*h*_) and A-type K^+^ current (*I*_*KA*_) in excitatory rhythm-generating interneurons (Zhang et al., 2015; Picton et al., 2018). *I*_*h*_ plays an important role in the functioning of rhythmic networks in both invertebrate (Angstadt and Calabrese, 1989; Peck et al., 2006) and vertebrate systems (Takahashi, 1990; Thoby-Brisson and Ramirez, 2000; Kjaerulff and Kiehn, 2001; Butt et al., 2002; Smith and Perrier, 2006; Picton et al., 2018; Sharples and Miles, 2021).

The circuit dynamics that produce episodic and continuous outputs and the mechanisms that drive transitions between these patterns are less clear. In many species, diverse outputs from motor circuits are produced through a combination of dedicated and multifunctional circuit elements that possess tunable properties (Meech and Thomas, 1977; Berkinblit et al., 1978; Mackie and Meech, 1985; Ramirez and Pearson, 1988; Getting, 1989; Berkowitz, 2002; Li et al., 2007; Gutierrez et al., 2013; Gutierrez and Marder, 2014; Parker et al., 2018). In support of a multifunctional circuit concept, we previously described a dynamic mechanism and developed a model of a single half-center oscillator (HCO) that could produce two rhythmic patterns with very distinct cycle periods (CPs): a slow locomotor-like pattern (∼1 s) and a fast paw-shake-like pattern (∼0.1 s) (Bondy et al., 2016; Parker et al., 2018).

Here, we explored cellular dynamics that enable a single CPG to produce very slow episodic patterns of rhythmicity (*episode* cycle *period*, EP ∼ 50 s) and identified changes in intrinsic biophysical properties that elicit transitions to faster continuous rhythmicity (*continuous* bursting cycle *period* BP ∼ 1 s). We adapted our established biophysical model (Parker et al., 2018) to produce episodic and continuous patterns of rhythmic activity, similar to patterns previously observed in the isolated neonatal rodent spinal cord *in vitro* (Barbieri and Nistri, 2001; Marchetti and Nistri, 2001; Gozal et al., 2014; Sharples and Whelan, 2017). We then compared the contribution of key intrinsic properties predicted by the model to the generation of episodic rhythmicity elicited by dopamine in experiments on isolated neonatal mouse spinal cords. This preparation offers a convenient means to study these dynamics because both episodic and continuous patterns can be elicited (Sharples and Whelan, 2017), and its cellular properties can be readily manipulated. Our complementary approaches provide novel insights into how CPGs produce multiple rhythmic patterns and transitions between them. Some of these results have been presented previously in abstract form (Vargas and Cymbalyuk, 2019).

## Results

### A Biophysical Model That Generates Episodic Rhythmicity

**FIGURE 1:**
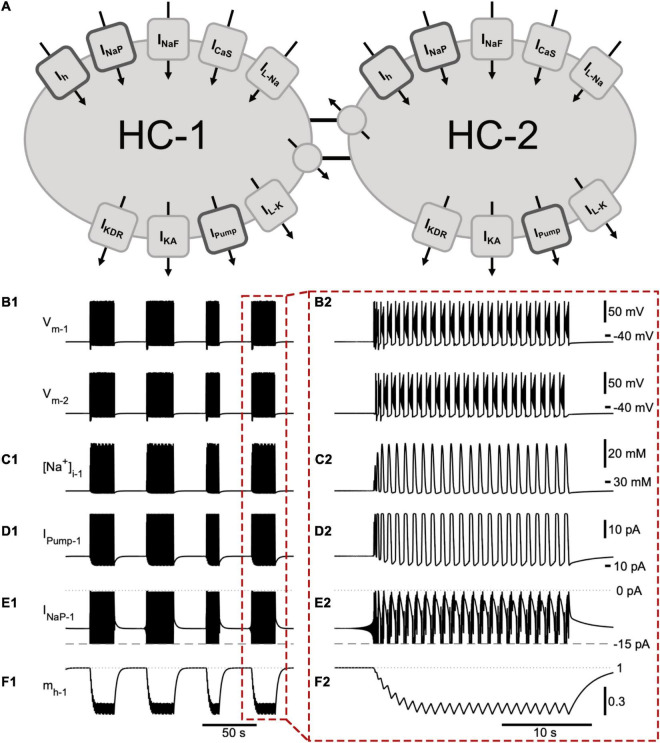
A half -center oscillator model produces episodic bursting activity. **(A)** Schematic representation of the model showing all membrane currents for the two mutually inhibitory cells of the half-center oscillator (HCO). Inward currents include the slow calcium current (*I*_*CaS*_), fast Na^+^ current (*I*_*NaF*_), persistent Na^+^ current (*I*_*NaP*_), Na^+^ leak current (*I*_*L–Na*_), and a hyperpolarization-activated cation current (*I*_*h*_). Outward currents are the potassium leak current (*I*_*L–K*_), delayed rectifier potassium current (*I*_*KDR*_), A-type potassium current (*I*_*KA*_), Na^+^/K^+^ pump current (*I*_Pump_), and inhibitory synaptic current (*I*_*Syn*_). **(B)** The membrane potential of the two model cells (*V*_*m*–1_ and *V*_*m*–2_) engage in episodic bursting activity **(B1)** and burst in alternation within an episode **(B2)**. The model is entirely symmetric, so the time course of a few important state variables and currents are shown for only one neuron (neuron 1): **(C1,C2)** intracellular Na^+^ concentration ([Na^+^]_*i1*_); **(D1,D2)** *I*_*Pump1*_; **(E1,E2)** persistent Na^+^ current (*I*_*NaP1*_). We clipped the trace of *I*_*NaP*_ below –15 pA in order to emphasize its contribution to intra-episode bursting. **(F1,F2)** activation of *I*_*h*_ (*m*_*h*_). The mechanism producing episodic bursting relies on *I*_*h*_, which slowly deactivates during the episode and activates between episodes promoting episode termination **(F2)**. A single episode enclosed in a red dashed box in **(B1–F1)** is expanded in **(B2–F2)**.

We developed a computational model consisting of two neurons representing mutually inhibiting neuronal populations, an HCO (Figure 1). Our goal was to elucidate a mechanism that explains how a single HCO could generate episodic and continuous bursting patterns, and then verify this mechanism by comparing model results to experimental data. Our computational model exhibited episodic bursting at the chosen “canonical” parameter set, with an episode duration (ED) of about 20 s and an interepisode interval (IEI) of about 30 s. The intra-episode burst period (BP) increased throughout the beginning of an episode and then plateaued at around 1 s. Intra-episode burst duration (BD) plateaued at 0.44 s, and intra-episode interburst interval (IBI) plateaued at 0.60 s.

We suggest that the dynamics of episodic bursting in the neonatal mouse locomotor CPG are dependent on *I*_*h*_, *I*_*NaP*_, and *I*_Pump_. These three currents were active both between and during bursts (Figure 1). *I*_*h*_ is a mixed cation current and generates notable Na^+^ influx. We modeled the Na^+^ and K^+^ components of *I*_*h*_ separately, which thereby allowed us to evaluate the contribution of *I*_*h*_ to the intracellular Na^+^ concentration ([Na^+^]_*i*_). *I*_*h*_ and *I*_*NaP*_ are inward currents, they depolarize the cell, are voltage dependent, and generate an influx of Na^+^. *I*_Pump_ is an outward current activated by sufficiently high intracellular Na^+^ concentration. *I*_Pump_ hyperpolarizes the cell and generates outflux of Na^+^. These opposing interactions created interesting dynamics enabling continuous and episodic bursting regimes (Figure 1).

While individual spikes were mostly produced by *I*_*NaF*_, *I*_*KDR*_, and *I*_*KA*_, the burst generating mechanism of our model was based on the dynamics of activation and inactivation of *I*_*NaP*_ and *I*_*CaS*_, which supported intra-burst spiking activity and controlled the initiation and termination of individual bursts within an episode. These standard mechanisms and their effects on the basic characteristics of the intra-episode bursting are described in detail in previous publications (Bondy et al., 2016; Olypher et al., 2006; Parker et al., 2018, 2019). In addition, the termination of a burst was controlled by interaction of currents through intracellular Na^+^ concentration. *I*_*NaP*_ and *I*_*NaF*_ generated strong Na^+^ influx that raised [Na^+^]_*i*_ and correspondingly activated *I*_*Pump*,_ which generated a strong Na^+^ outflux. Thus, *I*_Pump_ also contributed to the termination of the burst by producing a strong outward current in response to high spiking activity (Figure 1). Notably, these inward and outward currents interacted through membrane potential and Na^+^ concentration. *I*_Pump_ remained active following burst termination, contributing to the hyperpolarization of the cell and, thus, to partial activation of *I*_*h*_ between the bursts (Figure 1F2). Within an episode, four currents including the outward *I*_Pump_, inward *I*_*CaS*_, *I*_*NaP*_, and *I*_*h*_ determined the interburst interval. We used these considerations of the biophysical mechanisms to adjust the model bursting properties to the experimentally recorded characteristics of bursting activity collected at the end of individual episodes.

### Key Currents for Producing Episodic Rhythmicity

**FIGURE 2:**
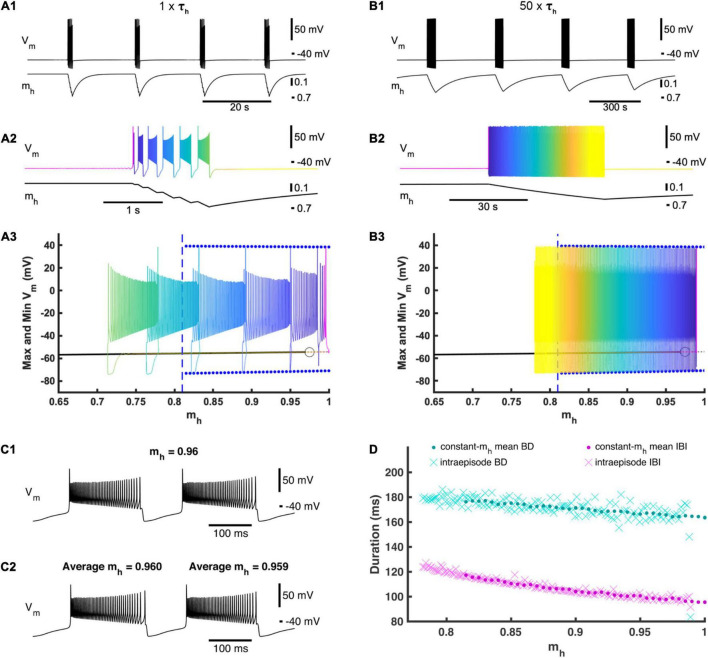
Activation and deactivation of *I*_*h*_ governs episodic bursting in the single cell model. **(A1)** A single cell in our full HCO model produces episodic activity when decoupled. **(A2)** Zooming in on intra-episode bursting in the single cell model. Color represents the phase within the episodic rhythm. **(A3)** Episodic activity of the full single-cell model is projected on the bifurcation diagram of the reduced constant-*m*_*h*_ model where *m*_*h*_ is treated as a parameter and varied. For each *m*_*h*_ parameter value, the reduced model maximum and minimum membrane potential values for the bursting regime are marked by blue dots, while the silent regime in the reduced model is marked by a black line. The silent regime loses stability at the subcritical Andronov–Hopf bifurcation marked by an open circle. The gray dashed line depicts an unstable silent regime, and the beginning of this unstable regime. The value of *m*_*h*_ where the bursting regime of the reduced model becomes unstable is marked by a vertical blue dashed line. The time course of the full single-cell model episodic activity can be seen by referencing the color map encoding phase defined in **(A2)**. **(B1)** Activity of the full single-cell model when τ*_*h*_* is scaled by multiplier factor 50 (50×τ*_*h*_*). **(B2)** Zooming in on a single episode to define the color map that represents the phase of this slower episodic rhythm. **(B3)** The slower episodic activity overlaid on the bifurcation diagram of the reduced constant-*m*_*h*_ model, where again the color map represents phase as defined in **(B2)**. **(C1)** Example of the reduced model with *m*_*h*_ = 0.96 to compare with **(C2)**. **(C2)** Zooming in on two consecutive bursts exhibited by the 50×τ*_*h*_* full single cell model, where the first of these two bursts have an average *m*_*h*_ value of 0.96 to be compared to **(C1)**. **(D)** Intra-episode burst characteristics from the 50×τ*_*h*_* model (cyan and magenta Xs) versus the average *m*_*h*_ across the corresponding burst cycle plotted with the mean burst duration (BD) and interburst interval (IBI) values of the reduced constant-*m*_*h*_ model (cyan and magenta dots) versus the value set for the parameter *m*_*h*_.

The mechanism producing episodic activity in our model was primarily based on *I*_*h*_ (Figures 1, 2). Having a time constant slower than 0.5 s, the activation of *I*_*h*_ (*m*_*h*_) was the slowest state variable of the system (Figure 1). Between episodes *I*_*h*_ activated and saturated at an *m*_*h*_ value close to 1. During the interepisode interval, inward and outward currents were roughly at balance. However, the opposing actions of *I*_*h*_ and *I*_*NaP*_ on one hand and *I*_Pump_ on the other created a very slow depolarization drift of the membrane potential leading to termination of the interepisode interval with a characteristic vibration in [Na^+^]_*i*_ and in membrane potential. These undamped oscillations slowly grew at the end of the interepisode interval until the peak of the oscillation surpassed threshold and the bursting phase then began. Within an episode, *I*_*h*_ partially deactivated during each burst and partially activated between bursts, but on average *I*_*h*_ progressively deactivated with each subsequent burst in an episode until it stabilized toward the end of the episode where inward and outward currents were in balance on average. At this phase, bursting within an episode was highly sensitive and variability in current levels from burst to burst is sufficient to trigger the end of the episode.

At the canonical parameter set, a single cell when decoupled also exhibited episodic activity. Compared to the HCO, the single cell episodic rhythm has about the same IEI of 17.3 s but ED is much shorter with a mean ED of only 1.27 s (Figures 2A1,A2). Similar to the HCO, the burst period increased throughout an episode, plateauing at the end of the episode. The average burst duration around the end of the episode decreased from the HCO model to 0.184 s, and the average interburst interval around the end of the episode decreased significantly to 0.127 s. To test the role of *m*_*h*_ in dynamics of episodic activity, we built a reduced single cell model where the variable *m*_*h*_ was held constant as a parameter (Figure 2A3). We varied *m*_*h*_ between 0 and 1 and observed only two regimes exhibited by the reduced model: (1) a stationary rest state corresponding to the interepisode interval and a continuous bursting regime corresponding to bursting activity within episodes. We found that the reduced model was silent for *m*_*h*_ values smaller than 0.815 (Figure 2A3) and exhibited only the bursting regime for *m*_*h*_ greater than 0.975. There was coexistence of the rest state and continuous bursting from 0.815 to 0.975 (Figure 2A3). Importantly, episodic activity did not occur when *m*_*h*_ was held constant, and therefore, the dynamics of *m*_*h*_ are necessary for episodic activity in this model. These findings suggest that the episodic bursting activity in the single cell could be understood as a second order bursting activity with bursts (episodes) of bursting activity. Then, the underlying mechanism is consistent with the elliptic bursting type (Bertram et al., 1995; Izhikevich, 2001) such that episodes of bursting activity correspond to bursts of spiking activity in a continuous bursting of elliptic type. According to its steady state activation curve, in the full model *m*_*h*_ grows when the cell is silent and decays on average when the cell is bursting.

To investigate this mechanism further, we overlaid the episodic activity of our full single cell model on the *m*_*h*_ bifurcation diagram for the reduced model (Figure 2A3). When the full model was in the silent phase, *m*_*h*_ slowly increased until it passes 0.975 where the silent regime loses stability through an Andronov–Hopf bifurcation in the reduced model, and *m*_*h*_ in the full model passed the bifurcation point of the fast subsystem and remained in the silent phase a little longer before jumping up to the bursting regime (Figures 2A1–A3). As bursting continued, *m*_*h*_ decreased (deactivated) during each burst until *m*_*h*_ crossed 0.815 where the bursting regime is no longer an attractor in the fast subsystem. Bursting continued beyond the transition value of *m*_*h*_ in the reduced model and *m*_*h*_ continued to decrease until the full model fell back to the silent regime (Figures 2A1–A3). We then increased the time constant of *m*_*h*_ by multiplying a scale factor of 50 to τ_*h*_ (Figure 2B). This slower model with increased τ_*h*_ exhibited a much longer ED of 45.0 s and a much longer IEI of 340 s, confirming the special leading role of the variable *m*_*h*_. The intra-episode burst characteristics did not change significantly. Also, the trajectory of the episodic bursting activity did conform better to the bifurcation diagram, although the bursting phase did still extend a little farther than in the reduced model (Figure 2B3). If *m*_*h*_ is the main driver of episodic activity, then the bursting regime of the reduced model should behave as a snapshot of the full model at the corresponding *m*_*h*_ value (Figure 2C) (Fenichel, 1979). We plotted mean bursting characteristics BD and IBI of the reduced model together with BD and IBI of the intra-episode bursts of the slower full model (50 × τ_*h*_) and found that they matched very closely (Figure 2D) indicating that the full model behaves as though *m*_*h*_ were being smoothly varied in the fast subsystem in a cyclic fashion with a period of 385 s.

### Model-Generated Episodic Rhythmicity Is Consistent With Spinal Rhythms Generated *in vitro*

**FIGURE 3:**
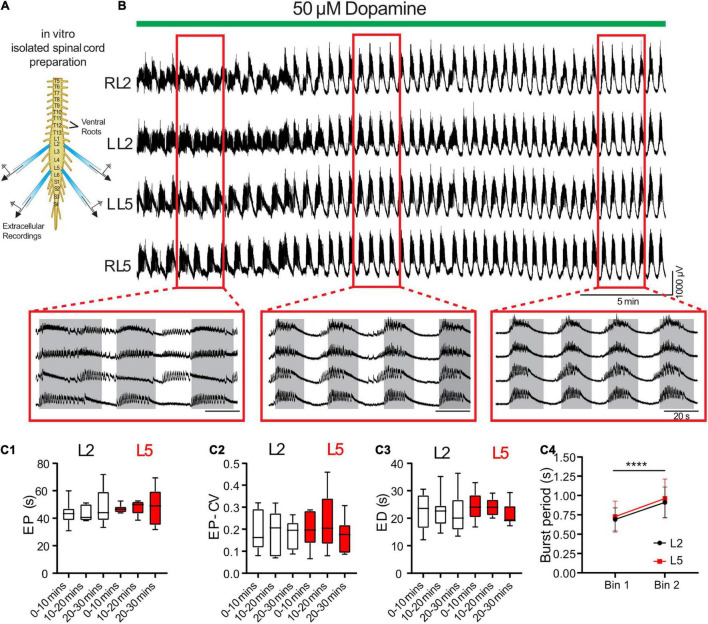
Dopamine elicits episodic bursting in isolated spinal cords of newborn mice. **(A)** Suction electrodes were used to record extracellular neurograms from left (L) and right (R) ventral roots of the second (L2) and fifth (L5) lumbar segments. **(B)** Dopamine applied at 50 μM (green bar) evoked episodic rhythmicity in lumbar spinal circuits. Expanded red boxes highlight changes in episode patterning across ventral roots over time. **(C)** From 0 to 30 min after dopamine application, episodes did not differ between L2 and L5 in episode period (EP: **C1**), episode period coefficient of variation (EP-CV: **C2**) or episode duration (ED: **C3**). Box and whisker plots display interquartile range (boxes), median (horizontal black lines), maximum, and minimum values in data range (whiskers). **(C4)** The burst period of the intra-episode rhythm increased between the first (bin 1) and second (bin 2) halves of an episode. Data in **(C4)** are presented as mean ± SD during the first (Bin 1) and second (Bin 2) half of each episode. Asterisks denote significance (****p < 0.0001, *n* = 8) from *post hoc* analyses following a 2-way ANOVA.

We validated predictions from the model using an isolated spinal cord preparation, in which we elicited episodic rhythmicity by bath application of dopamine (Figure 3; *n* = 8) (Sharples and Whelan, 2017). This preparation also allowed us to manipulate and assess the relative contribution of certain currents, which the model predicts play a key role in the rhythmic network activity. 50 μM dopamine elicited stable intervals of episodic activity with episode period (EP) = 48.5 ± 13.0 s, EP variability defined by the coefficient of variation (EP-CV) = 0.17 ± 0.07, ED = 21.5 ± 5.7 s, and IEI = 27.0 ± 14.1 s. Episodes in the L2 and L5 did not differ or vary over time in terms of EP [Figure 3C1; *F*_(2,14)_ = 3.4, *p* = 0.11], EP-CV [Figure 3C2; *F*_(2,14)_ = 0.7, *p* = 0.7], or ED [Figure 3C3; *F*_(2,14)_ = 0.28, *p* = 0.6]. Consistent with previous reports (Sharples et al., 2015; Sharples and Whelan, 2017), intra-episode bursting slowed over the duration of an episode and did not differ between L2 and L5 [Figure 3C4; *F*_(1,7)_ = 0.58, *p* = 0.5].

**FIGURE 4:**
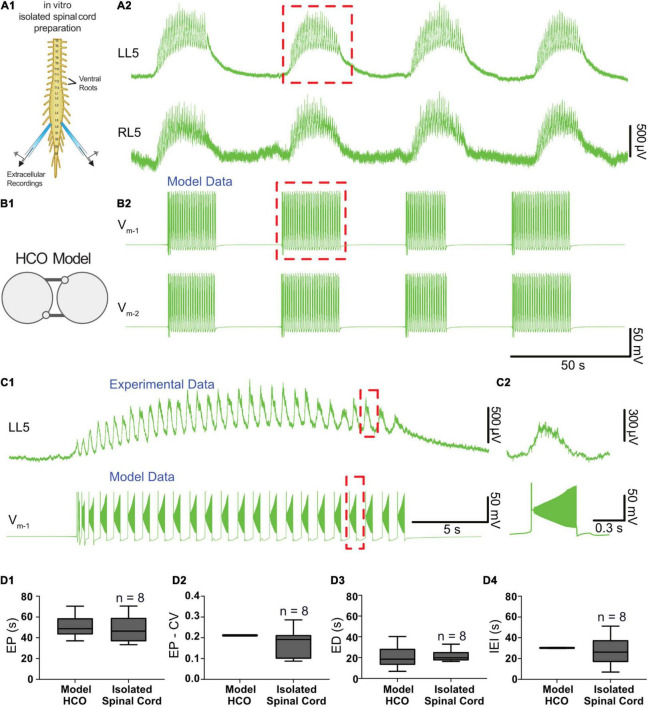
Temporal characteristics of dopamine-evoked episodic rhythmicity in the isolated spinal cord, reproduced by a biophysical model. **(A1)** Schematic showing recording setup used to obtain extracellular neurograms from isolated neonatal mouse spinal cords. **(A2)** Episodes of rhythmic activity generated by isolated spinal cords with dopamine (50 μM). Neurograms represent recordings from left and right L5 ventral roots. **(B1)** The half-center oscillator (HCO) model was tuned to produce episodic rhythmicity **(B2)**. **(C1)** Similar intra-episode rhythm features in a single episode of activity recorded experimentally (top trace) and from the model (lower trace) show bursting slowing down throughout the episode. **(C2)** The burst period and burst duration of a single intra-episode burst recorded experimentally (top trace) corresponds closely to a single burst within the model (lower trace), especially for the latter portion of the episode. **(D1)** Model-generated episodes (bottom trace) of activity match the recordings (top trace) in terms of episode period (EP). **(D2)** episode period variability (EP-CV), **(D3)** episode duration (ED), and **(D4)** interepisode interval (IEI). Box and whisker plots depict interquartile range (boxes), median (horizontal black lines), maximum, and minimum values in data range (whiskers).

We tuned a variety of parameters such that the model exhibited episodic bursting activity with temporal characteristics of EPs, EDs, and IEIs close to episodes generated by isolated spinal cords in response to 50 μM dopamine (Figures 3, 4A,B). We found a canonical parameter set that produced a mean EP = 51.1 ± 10.8 s, EP-CV = 0.211, ED = 20.8 ± 10.8 s, and IEI = 30.2 ± 0.3 s (Figure 4). With this parameter set, episodes generated by the model were not significantly different from those recorded *in vitro* from the spinal cord (*n* = 8) in terms of EP [Figure 4D1, *t*_(56)_ = 0.7, *p* = 0.5], EP variability [EP-CV; Figure 4D2, *t*_(7)_ = 0.6, *p* = 0.6], ED [Figure 4D3; *t*_(56)_ = 0.1, *p* = 0.9], or IEI [Figure 4D; *t*_(56)_ = 1.6, *p* = 0.1]. Furthermore, the model could reproduce intra-episode burst period dynamics observed *in vitro*, with an intra-episode burst period that started short and increased in length during the episode (Figures 4C1,C2). Intra-episode burst period in the model increases from 0.32 s for the first burst to 0.54 s for the second burst, and then increases steadily throughout the episode until it reaches 1.0 s for the last burst in the episode. In the experimental data, intra-episode burst period increased from 0.65 ± 0.2 s at the beginning of the episode to 0.9 ± 0.2 s at the end of the episode.

### Episodic Rhythmicity Depends on *I*_*h*_

We empirically identified ranges of parameters around their canonical values (one at a time) within which our HCO model exhibited episodic activity. Changing the parameters of *I*_Pump_ and *I*_*h*_ moved the system into and out of the zone supporting episodic bursting. In each case of specific parameter variations, we compared changes predicted by the model with experimental data from the isolated spinal cord preparation. We found that the parameters of *I*_Pump_, *I*_*h*_, and the inhibitory synaptic current (*I*_*Syn*_) required careful adjustment to fit the temporal characteristics of experimental data.

**FIGURE 5:**
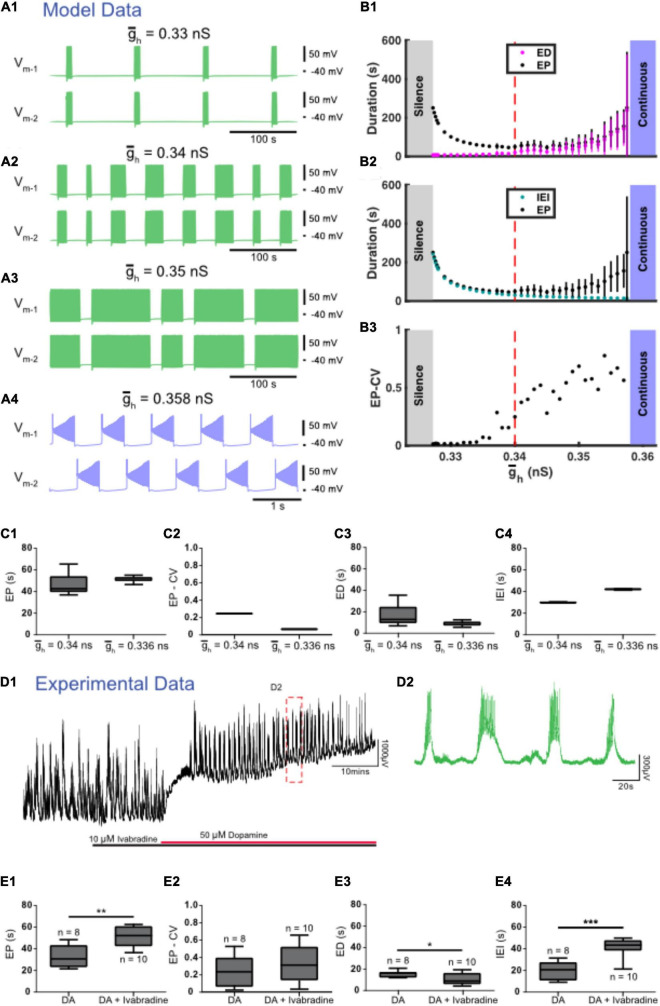
Regulation of ${\bar{g}}_{h}$ accurately predicts the effects of experimental blockade of *I*_*h*_ with ivabradine. **(A)** Examples of various types of activity produced at different values of the maximal conductance of *I*_*h*_ (${\bar{g}}_{h}$) are depicted. Episodic activity occurs at ${\bar{g}}_{h}$ = 0.33 nS **(A1)**, at ${\bar{g}}_{h}$ = 0.34 nS (canonical) **(A2)**, and at ${\bar{g}}_{h}$ = 0.35 nS **(A3)**, where episode duration (ED) increased and interepisode interval (IEI) decreased as ${\bar{g}}_{h}$ increased. **(A4)** Continuous bursting occurred at ${\bar{g}}_{h}$ = 0.358 nS. **(B)** Episodic activity properties and a transition to continuous activity depend on ${\bar{g}}_{h}$. **(B1)** Mean episode period (EP) (black dots) and mean ED (magenta dots) increased exponentially as ${\bar{g}}_{h}$ increased. **(B2)** Mean IEI (cyan dots) increased exponentially as ${\bar{g}}_{h}$ decreased. **(B3)** Mean EP-CV (black dots) increased with g_*h*_. **(C)** Box and whisker plots depict interquartile range (boxes), median (horizontal black lines), maximum and minimum values in the data range (whiskers). **(C1)** When ${\bar{g}}_{h}$ was decreased, EP increased, **(C2)** the coefficient of variation of EP (EP-CV) decreased, and **(C3)** ED decreased and IEI increased **(C4)**. **(D)** Extracellular neurograms **(D1)** recorded from a single ventral root show episodic rhythmicity evoked by dopamine (red bar) in the presence of the HCN channel blocker ivabradine (10 μM, black bar). Panel **(D2)** expands the inset in **(D1)**. **(E)** Ivabradine increased the EP **(E1)**, did not affect EP-CV **(E2)**, reduced ED **(E3)**, and increased the IEI **(E4)** of dopamine-evoked rhythmicity. Box and whisker plots depict interquartile range (boxes), median (horizontal black lines), maximum, and minimum values in the data range (whiskers) for episodic rhythms evoked by dopamine alone (*n* = 8), or in the presence of ivabradine (*n* = 10) in separate sets of experiments; asterisks indicate significance of unpaired *t*-tests (*p < 0.05; ***p < 0.001).

We found three zones of parameter space when *I*_*h*_ was manipulated. The canonical value for the maximal conductance of *I*_*h*_ (${\bar{g}}_{h})$ was set to 0.34 nS. When ${\bar{g}}_{h}$ was decreased from this canonical value to simulate partially blocking *I*_*h*_, ED decreased and IEI increased (Figures 5A1,A2,B,C). Similar to experiments, IEI increased faster than ED causing EP to also increase (Figure 5B). When ${\bar{g}}_{h}$ was decreased further to 0.327 nS, activity switched to silence. On the other hand, when ${\bar{g}}_{h}$ was increased from 0.34 nS ED increased and IEI decreased until ${\bar{g}}_{h}$ reached 0.358 at which point activity switched to continuous bursting (Figures 5A3,A4,B,C).

We next tested the contribution of *I*_*h*_ to the control of dopamine-evoked episodes with the HCN channel blocker ivabradine, which dose-dependently decreased the duration of episodes evoked by dopamine (IC_50_ = 1.6 μM; 1 nM: ED = 14.7 ± 2 s, *n* = 4; 10 nM: 15.2 ± 2.3 s, *n* = 4; 100 nM: 15.4 ± 2.2 s, *n* = 4; 1 μM; 15.7 ± 2.2 s, *n* = 4; 10 μM: 10.7 ± 5.1 s, *n* = 10; 20 μM: 12 ± 1.2 s, *n* = 4), and consistent with the model, suppressed episodic activity at the highest concentrations tested (40–100 μM; *n* = 4). Episodic activity elicited by dopamine in the presence of 10 μM ivabradine (Figures 5D,E; *n* = 10 preparations) had a significantly longer EP [*t*_(16)_ = 3.949, *p* = 0.0011, DA: 32.8 ± 10.5; DA + ivabradine: 51.7 ± 9.8 s], with no change in EP-CV [*t*_(16)_ = 1.034, *p* = 0.3167, DA: 0.25 ± 0.18; DA + ivabradine: 0.34 ± 0.21 s], shorter ED [*t*_(16)_ = 2.332, *p* = 0.0331, DA: 15.4 ± 3; DA + ivabradine: 10.7 ± 5.1 s], and longer IEI [*t*_(16)_ = 5.484, *p* = 0.0001; DA: 19.9 ± 8.5; DA + ivabradine: 41.7 ± 8.3 s]. The effects of ivabradine on episodic activity *in vitro* are consistent with observations from the model.

We also tested the contribution of *I*_*h*_ to the control of episodic activity with an additional, and more commonly deployed HCN channel blocker, ZD7288. In the presence of ZD7288, dopamine (50 μM) depolarized the ventral root neurogram (Supplementary Figure 1A1), followed by the emergence of erratic episodic rhythmic bursting that resembled patterns observed during spontaneous activity (Supplementary Figure 1A2). Compared to episodes elicited by preparations with dopamine alone (*n* = 16), dopamine-evoked episodes in the presence of ZD7288 (*n* = 21) had longer EPs [*t*_(35)_ = 2.131, *p* = 0.0402, DA: 33.6 ± 8.4; DA + ZD7288: 40.2 ± 10 s; Supplementary Figure 1B1], were more irregular [EP-CV; *t*_(35)_ = 3.196, *p* = 0.0029, DA: 0.24 ± 0.16; DA + ZD7288: 0.39 ± 0.12 s; Supplementary Figure 1B2], with no changes in EDs [*t*_(35)_ = 1.284, *p* = 0.2077, DA: 17 ± 3.3; DA + ZD7288: 19 ± 5.6 s; Supplementary Figure 1B3], or IEI [*t*_(35)_ = 1.129, *p* = 0.2666; DA: 18 ± 7.5 s; DA + ZD7288 21.2 ± 8.9 s; Supplementary Figure 1B4].

### Modulation of *I*_*Pump*_ Leads to Transitions Between Episodic and Continuous Bursting

**FIGURE 6:**
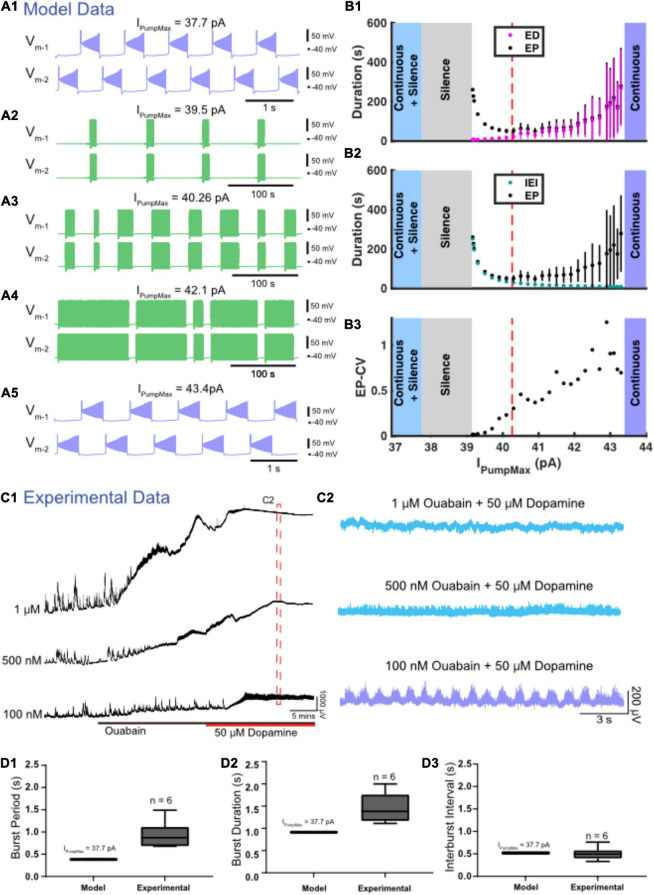
Variation in *I*_PumpMax_ modulates episodic bursting properties and produces transitions between episodic and continuous rhythmicity. **(A)** Examples of various activity types produced at different values of the maximal pump strength (*I*_PumpMax_) are depicted. **(A1)** Continuous bursting occurs at *I*_PumpMax_ = 37.7 pA. **(A2)** Episodic activity occurs at *I*_PumpMax_ = 39.3 pA with long interepisode intervals (IEIs) and short episode durations (EDs). **(A3)** At *I*_PumpMax_ = 40.26 pA (canonical), episodic activity occurs with moderately variable EDs. **(A4)** At *I*_PumpMax_ = 42.1 pA, episodic activity occurs with highly variable EDs and short IEIs. **(A5)** Continuous bursting occurs at *I*_PumpMax_ = 43.4 pA. **(B)** Graphs depict the relationship between *I*_PumpMax_ and episode parameters as well as transitions between regimes. **(B1)** EP (black dots), EP variability (black bars) and ED (magenta dots) and ED variability (magenta bars) are shown versus *I*_PumpMax_. **(B2)** EP (black dots) and IEI (cyan dots) are shown versus *I*_PumpMax_. **(B3)** EP-CV versus *I*_PumpMax_. **(C1)** Extracellular neurograms of spontaneous activity recorded in DC from ventral roots during application of 1 μM (*n* = 6), 500 nM (*n* = 7), and 100 nM (*n* = 6) of ouabain (black bar) and subsequent application of dopamine (50 μM: red bar). **(C2)** Neurograms displaying network activity elicited by dopamine in the presence of different concentrations of ouabain highlighted in **(C1)**. **(D)** Box and whisker plots depict interquartile range (boxes), median (horizontal black lines), maximum and minimum values in the data range (whiskers) for burst period (BP: **D1**), burst duration (BD: **D2**), and interburst interval (IBI: **D3**) during continuous bursting generated by the Model HCO with *I*_PumpMax_ = 37.7 pA compared to continuous bursting generated by isolated spinal cords (Experimental) in the presence of 50 μM dopamine and 100 nM ouabain.

Four different zones of parameter space were found when the maximal strength of *I*_Pump_ (*I*_PumpMax_) was varied (Figures 6A,B). The model exhibited episodic bursting within the range of *I*_PumpMax_ values between 39.1 and 43.4 pA (Figures 6A2–A4,B). When *I*_PumpMax_ was decreased from that episodic regime, the model fell silent at *I*_PumpMax_ = 39.1 pA, and bistability of silence and continuous bursting presented when *I*_PumpMax_ was decreased past 37.7 pA (Figures 6A1,B). As *I*_PumpMax_ was decreased, the continuous bursting became more plateau-like in this bistable regime. The model fell silent again when *I*_PumpMax_ was decreased to 32.2 pA (Figure 6B). When *I*_PumpMax_ was increased from the episodic bursting regime, activity switched to another continuous bursting regime at *I*_PumpMax_ = 43.4 pA (Figures 6A2–A5,B). Thus, the model produced episodic bursting within a range of *I*_PumpMax_ values and could transition from episodic bursting to continuous bursting at either end of the range (Figures 6A,B).

As *I*_PumpMax_ increased within the range supporting episodic bursting, ED and EP increased exponentially (Figure 6B1). The rate of change for ED and EP became particularly prominent when *I*_PumpMax_ was increased above 42.0 pA, where changes in *I*_PumpMax_ had a greater effect on ED, relative to its effect on IEI (Figures 6B1,B2). On the other hand, as *I*_PumpMax_ decreased toward the lower boundary between episodic activity and silent activity (39.1 pA), IEI increased exponentially, while ED decreased only slightly. The opposite trends of ED and IEI cause EP to form a U-shape (Figures 6B1,B2). The variability of EP increased significantly as *I*_PumpMax_ increased (Figure 6B3), mainly due to an increase in the variability of ED. The continuous bursting found in our model at lower values of *I*_PumpMax_ is consistent with experimental results obtained when the pump current was blocked with ouabain (Figures 6D1,D2).

We validated our model’s predictions by testing the effect of varying *I*_Pump_ strength using different concentrations of ouabain to inhibit the sodium pump and subsequently examine effects on the rhythmic activity in the isolated spinal cord. Ouabain has dose-dependent effects on *I*_Pump_, preferentially disrupting the α_3_ subunit and dynamic pump function at low concentrations while impairing the α_1_ subunit at higher concentrations (Lichtstein and Rosen, 2001). Prior to application of dopamine, ouabain concentrations of 1 μM (Figure 6C; *n* = 6; mean ± SD Δ DC potential = 3433 ± 1897 μV) and 500 nM (*n* = 7; mean ± SD Δ DC potential = 868 ± 547 μV) dose-dependently depolarized ventral root DC potentials [Figures 6B1,C1: *F*_(2,16)_ = 13.7, *p* = 0.0003] and suppressed spontaneous network activity [Figure 6C1: *F*_(2,16)_ = 9.0, *p* = 0.002]. Subsequent application of dopamine when 500 nM or 1 μM of ouabain was present further depolarized the ventral roots but did not elicit any superimposed rhythmicity (Figure 6C2; top two traces in blue), consistent with the silent state predicted by the model at the lowest *I*_PumpMax_ strengths. As predicted by the model, reducing ouabain concentration to 100 nM produced continuous rhythmic bursting in the presence of dopamine with burst metrics within a range similar to those generated by the model (Figures 6C1,C2). Continuous bursting elicited by dopamine in the presence of 100 nM of ouabain had a mean burst cycle period (BP) of 1.45 ± 0.37 s, a BP-CV of 0.22 ± 0.07, BD of 0.93 ± 0.33 s, IBI of 0.50 ± 0.2 s, and were within range of the burst characteristics generated by the HCO model with *I*_PumpMax_ = 37.7 pA (Figures 6D1–D3). Episodic bursting returned in three of six preparations following a wash with 50 μM dopamine in regular aCSF (not shown).

**FIGURE 7:**
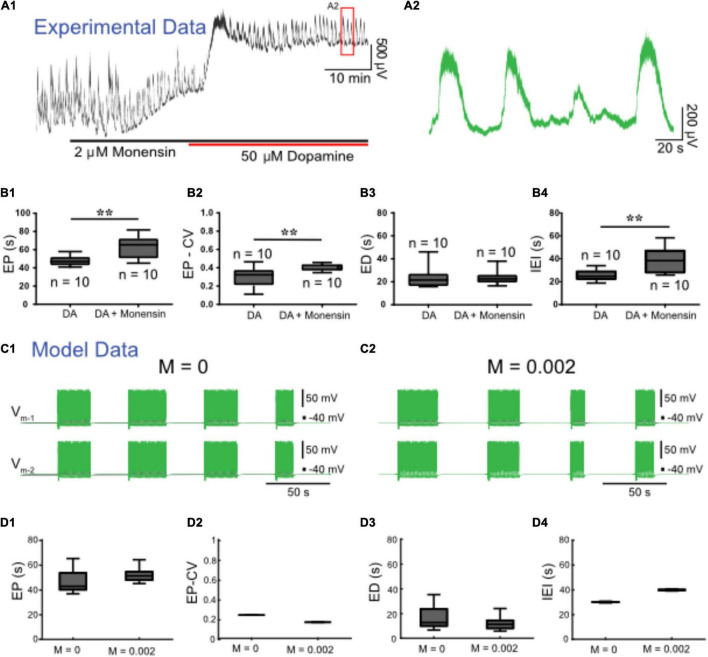
Monensin increases episode period and interepisode interval in both modeled and experimental data. **(A1)** Extracellular neurograms recorded from ventral roots with bath application of 2 μM monensin (black bar along the *x*-axis) followed by 50 μM dopamine (red bar along *x*-axis). **(A2)** Expanded segment from inset (red box) in **(A1)**. **(B1)** Episode period (EP) increases with monensin **(B2)** but becomes more variable, **(B3)** while episode duration (ED) is maintained, and **(B4)** interepisode interval (IEI) is increased. Asterisks denote significance level following unpaired *t*-tests (DA: *n* = 10; DA+ Monensin: *n* = 10). We simulated monensin application in the model HCO by increasing the influx of intracellular sodium [Na^+^]_*i*_ using a monensin rate constant (M). Changing this rate constant from M = 0 **(C1)** to M = 0.002 **(C2)** in the model reproduced most of the effects of monensin on isolated spinal cords, leading to **(D1)** an increase in EP, **(D2)** decrease in EP-CV, **(D3)** no change in ED, and **(D4)** an increase in IEI.

We next set out to upregulate *I*_Pump_ with monensin, an antibiotic that acts as a Na^+^-H^+^ antiporter, leading to increased [Na^+^]_*i*,_ and, thus, indirectly increasing *I*_Pump_ activity (Kueh et al., 2016; Picton et al., 2017). Prior to application of dopamine, 2 μM monensin (*n* = 12) reduced spontaneous bursting activity. Subsequent application of 50 μM dopamine with monensin depolarized the ventral root potentials and produced episodic bursting in 10 preparations (Figure 7A). In 2 preparations we observed a depolarization following DA application, but no episodic rhythmic bursting was evoked. Compared to episodes evoked by dopamine alone (*n* = 10), episodes evoked by dopamine in the presence of monensin (*n* = 10) had a longer EP [*t*_(18)_ = 3.411, *p* = 0.0003, DA: 47.6 ± 5.8; DA + monensin: 62.5 ± 12.5 s], were more irregular EP-CV [*t*_(18)_ = 2.927, *p* = 0.009, DA: 0.29 ± 0.11; DA + monensin: 0.4 ± 0.03], had a longer IEI [*t*_(18)_ = 3.511, *p* = 0.0025, DA: 25.1 ± 4.8; DA + monensin: 38.8 ± 11.3 s], with no change in ED [*t*_(18)_ = 0.1651, *p* = 0.8707, DA: 23.2 ± 9; DA + monensin: 23.8 ± 6.6 s] (Figure 7B). Consistent with experimental findings, applying ‘monensin’ in the HCO model, represented by a monensin rate constant *M* = 0.002, led to an increase in EP and IEI, no significant change in ED, but decreased EP-CV (Figures 7C,D). Mean EP increased to 55.8 ± 9.2 s, EP-CV decreased to 0.165, and IEI increased to 39.9 ± 0.6 s.

## Discussion

**FIGURE 8:**
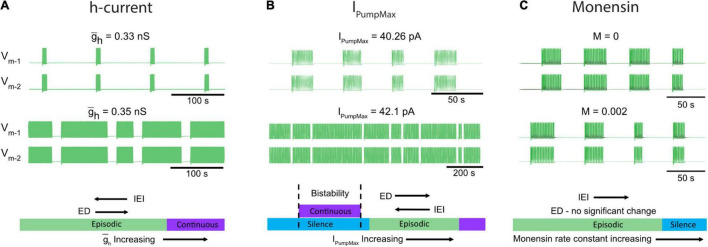
Neuromodulators evoke diverse rhythmic outputs by moving the network through an excitation-based parameter space by altering *I*_PumpMax_, and ${\bar{g}}_{h}$. **(A)** Two traces showing behavior of the model while altering the *h*-current. **(B)** Changes in episodic activity following manipulation of *I*_PumpMax_ and **(C)** model output before and after the introduction of a monensin factor. The bottom schematics **(A–C)** summarize the changes in rhythmic characteristics following manipulation of the *h*-current and *I*_PumpMax_, or through introduction of a monensin factor.

Spinal circuits produce a diverse array of rhythmic motor outputs, a subset that can be generated at birth (Whelan et al., 2000). Episodic activity has been reported in developing spinal circuits for a range of species (Combes et al., 2004; McDearmid and Drapeau, 2006; Gabriel et al., 2011; Wiggin et al., 2012; Gozal et al., 2014; Currie et al., 2016; Sharples and Whelan, 2017; Mahrous and Elbasiouny, 2018; Picton et al., 2018; Kondratskaya et al., 2019). Episodic patterns of rhythmic activity can be generated by the *in vitro* perinatal mouse spinal cord, and are not specific to dopamine (Sharples and Whelan, 2017; Sharples et al., 2020), but can also be elicited by trace amines (Gozal et al., 2014) or activation of tachykinin receptors (Barbieri and Nistri, 2001; Marchetti and Nistri, 2001), or in juvenile mouse isolated spinal cords (Mahrous and Elbasiouny, 2018). Further, transitions between episodic and continuous patterns can be induced by manipulating neuromodulatory tone or excitability (Sharples and Whelan, 2017). Here, we focused on cellular mechanisms that produce episodic and continuous patterns of rhythmic activity and the mechanisms that govern transitions between them (Figure 8). To our knowledge, we developed the first biophysical model that can produce both episodic and continuous patterns of bursting activity and qualitatively investigated how key network and cellular properties contribute to their generation in isolated spinal cords. We subsequently tuned model parameters to produce episodic bursting with temporal features that are remarkably consistent with those elicited in isolated spinal cords by dopamine. The model was a single CPG composed of two inhibitory half-centers that could adjust parameters of episodic bursting and switch output modes through modulation of *I*_Pump_ and *I*_*h*_. We tuned these parameters applying insights based on studies on the dynamic roles of *I*_Pump_ and *I*_*h*_ in the regulation of the leech heartbeat (Tobin and Calabrese, 2005; Kueh et al., 2016) and *Xenopus* tadpole locomotor CPGs (Zhang and Sillar, 2012; Zhang et al., 2015; Picton et al., 2018). The simplified model does not provide quantitative estimation of these parameters. We used the model to qualitatively describe temporal changes of the episodic bursting pattern and its transitions into either silence or continuous bursting representing experimental manipulations of *I*_Pump_ and *I*_*h*_.

The model predicted that decreasing the strength of *I*_*h*_ would decrease ED and increase IEI and if decreased further would eventually lead to silence. Consistent with this finding, blocking *I*_*h*_ with ivabradine reduced ED and increased IEI evoked by dopamine *in vitro* (Figure 8A). The model also predicted that either up or down-regulation of *I*_PumpMax_ would produce a transition to continuous bursting primarily through regulation of the interepisode interval. This finding is supported by our *in vitro* pharmacological experiments where continuous bursting or silence was produced when *I*_Pump_ was dose-dependently reduced with ouabain, and increasing *I*_Pump_ with monensin increased the IEI (Figures 8B,C). Collectively, our results demonstrate that multiple pathways can lead to transitions between episodic and continuous rhythmic patterns (Figure 8). This finding is consistent with studies modeling the stomatogastric ganglion (STG), which demonstrated that disparate circuit properties produced similar outputs (Prinz et al., 2004) and led to transitions between circuit outputs (Gutierrez et al., 2013; Gutierrez and Marder, 2014). It is therefore likely that other properties that we did not study could also contribute to the generation of episodic bursting and their modulation lead to transitions between episodic and continuous bursting.

### Dynamic Mechanisms Lead to Transitions Between Episodic and Continuous Rhythmic Activity in a Single Developing Central Pattern Generator

We provide an analysis of model activity when each element in our model is individually manipulated. We find parallel changes in biological network behavior when these elements are individually modulated pharmacologically. While our model was not developed to simulate the modulatory effects of dopamine on spinal neurons, the properties of the neurons within our model are in line with reported effects of dopamine on spinal neurons. For example, the pump-mediated ultraslow after hyperpolarization (usAHP) is expressed in mouse spinal interneurons and motoneurons at early postnatal stages and is increased by dopamine (Picton et al., 2017). Of the properties established in our model, we found that *I*_Pump_ plays a critical role in generating episodic bursting with manipulations in *I*_PumpMax_ leading to switching between episodic and continuous bursting through modulation of the IEI. In line with our model, blocking the pump with low concentrations of ouabain (100 nM), which may select toward blocking the α_3_ subunit of the pump (Lichtstein and Rosen, 2001), promotes the transition from episodic to continuous bursting. In contrast, higher concentrations of ouabain (0.5–1 μM) inhibited rhythmic activity in the presence of dopamine and is consistent with the silent output predicted by the model at the lowest *I*_PumpMax_ strengths. Manipulating the pump by increasing intracellular sodium concentration experimentally with monensin caused an increase in EP and IEI while ED remained constant. This is consistent with modeled data where *I*_Pump_ was increased indirectly through the introduction of a ‘monensin factor.’

Interestingly, increasing *I*_PumpMax_ in the model also prompted a transition from episodic to continuous bursting. Increased pump activity may produce transitions from episodic to continuous locomotor behaviors observed during development in fish and tadpoles (Müller et al., 2000; Buss and Drapeau, 2001; Gabriel et al., 2011; Lambert et al., 2012; Hachoumi and Sillar, 2020). However, our work raises the possibility that a reduction in dynamic pump function may also generate such transitions. Thus, modulating the pump’s function may change the mode of operation during development (Gonzalez-Islas et al., 2020), in addition to dynamically regulating locomotor behavior.

Rhythm-generating interneurons also often express the mixed cation-conducting current *I*_*h*_ (Kiehn et al., 2000; Wilson et al., 2005; Dougherty and Kiehn, 2010; Borowska et al., 2013), which is typically activated at hyperpolarized potentials and supports escape from inhibition, and in turn facilitates burst initiation. Importantly, our model is critically dependent on the dynamics of *h*-current, with *I*_*h*_ being largely active at the silent interepisode interval, slowly deactivating over the duration of an episode, and subsequently producing episode termination. In line with our model, a resting I_*h*_ is expressed in a subset of spinal motoneurons during later stages of postnatal development in mice (Sharples and Miles, 2021), in addition to glutamatergic rhythm-generating interneurons (dINs) of *Xenopus* tadpoles, where it opposes and mitigates the hyperpolarizing influence of *I*_Pump_ on the membrane potential (Picton et al., 2018). Furthermore, multiple modulators, including dopamine and serotonin, have been reported to depolarize the activation voltage of *I*_*h*_ in neurons (Kjaerulff and Kiehn, 2001; Ballo et al., 2010), and *I*_*h*_ can be active near the rest potential. *I*_*h*_ also interacts with *I*_Pump_ in leech heartbeat CPG neurons, which accounts for the paradoxical speeding up of the rhythm when *I*_Pump_ is stimulated experimentally with monensin (Kueh et al., 2016). Our data is consistent with these studies; we found that increasing both *I*_PumpMax_ and *I*_*h*_ in the model led to a transition from episodic to continuous rhythms. We used two HCN channel blockers, ZD7288 and ivabradine, to determine the contribution of *I*_*h*_ to the generation of episodic activity produced by dopamine in isolated spinal cords. Whilst ZD7288 did affect episodic activity, it produced a pattern that no longer represented episodic activity elicited by dopamine under control conditions, but instead produced motor patterns that are consistent with spontaneous activity generated in the absence of stimulation (Dalrymple et al., 2019). ZD7288 has been reported to block other ion channels including Cav1.3 and NaV1.4 (Sánchez-Alonso et al., 2008; Wu et al., 2012), which contribute to T-type calcium currents and persistent sodium currents, respectively, and could also control episodic activity. We therefore chose to examine ivabradine, a newer *I*_*h*_ blocker. Ivabradine produced qualitatively different effects on the rhythm compared to ZD7288, namely while episodic patterns were affected, the pattern of slow episodes with superimposed fast bursting found under control conditions was retained. Ivabradine increased the EP, decreased ED, and increased IEI, which is consistent with what we find when decreasing ${\bar{g}}_{h}$ in the model. Alternatively, ivabradine has been shown to reduce *M*-currents (Hsiao et al., 2019), which could also lead to changes in episodic bursting (Verneuil et al., 2020). Our results support a role for *I*_*h*_ in the control of episodic activity – with consistent effects on ED and IEI observed. It is unclear whether ivabradine selectively or uniformly targets each of the HCN subunit isoform channels (Emery et al., 2012; Tanguay et al., 2019). This is an area for further investigation.

Our data support multiple mechanisms that lead to transitions between episodic and continuous bursting patterns with modulation of *I*_*h*_ affecting IEI and ED while *I*_Pump_ controls the IEI. This work extends the knowledge on the pump’s diverse functions in the dynamic regulation of neuronal excitability described in a wide range of model systems (Ballerini et al., 1997; Pulver and Griffith, 2010; Zhang and Sillar, 2012; Kueh et al., 2016; Picton et al., 2017, 2018; Zhang et al., 2017; Tiwari et al., 2018; Gerkau et al., 2019). Given that *I*_Pump_ and *I*_*h*_ are important for the dynamic activity-dependent regulation of neural networks, and in some cases, producing short term motor memory (STMM) (Picton et al., 2018), an interesting area for future investigation would be to determine how modulation of these properties can adjust network behaviors such as STMM [as discussed by Hachoumi and Sillar (2020)].

### Flexible Outputs Produced by a Single Multifunctional Central Pattern Generator

An important question that remains only partially resolved is whether spinal circuits produce diverse rhythmic activities by recruiting multiple dedicated rhythm generating circuits or through the modulation of a single multifunctional CPG? The latter appears to be true in many vertebrate systems and has been well described in invertebrate CPGs (Blitz et al., 1999; Briggman and Kristan, 2006). For example, in the turtle, scratching and swimming movements are produced by a combination of overlapping and dedicated populations of spinal interneurons (Berkowitz, 2002; Hao et al., 2011; Snyder and Rubin, 2015). Similar results have been found for swimming and struggling motor patterns in both *Xenopus* tadpoles (Soffe, 1993; Li et al., 2007) and larval zebrafish (Liao and Fetcho, 2008). Overlapping populations of spinal interneurons appear to contribute to the generation of walking and paw-shaking in cats (Carter and Smith, 1986; Barbeau and Rossignol, 1990), and our previous computational models proposed a multifunctional CPG with dedicated roles for calcium and sodium conductances in the generation of these two rhythmic motor patterns (Parker et al., 2018, 2019). The episodic and continuous bursting modes that we describe here could serve as a new system to study how mammalian spinal circuits produce diverse rhythmic activities. Whether these two patterns are produced by dedicated or overlapping neural elements in the spinal cord remains to be determined.

Dialog between experimental and computational systems offers insight into mechanisms of multifunctionality. Computational models inspired by the organization of the STG have elegantly demonstrated that degenerate mechanisms in electrical and chemical inhibitory synapses can produce pattern switching in a small multifunctional circuit (Gutierrez et al., 2013). Our computational model, composed of an inhibitory HCO, suggests that modulation of key intrinsic properties can lead to multifunctionality within a single circuit. Indeed our HCO model represents a fundamental element of current computational models of spinal CPGs (Yakovenko et al., 2005; Rybak et al., 2006; Ausborn et al., 2018; Shevtsova et al., 2020) and may serve as a key locus to produce flexible rhythmic outputs. Therefore, this work offers insight into how spinal circuits and computational models of locomotor CPGs can produce adaptable locomotor output with the integration of key intrinsic properties as targets for neuromodulators serving as a basis for their control. Our focus here is on the generation of episodic locomotor activity which contains both slow episodic and fast locomotor components and we show that a simplified HCO can produce both elements. While we show that *I*_*h*_ and *I*_Pump_ in an inhibitory circuit are key properties for episodic and continuous rhythms, there are likely others.

### Limitations of the Model

Our model is a basic HCO consisting of two units with mutual inhibitory synaptic connectivity. We realize that the vertebrate spinal locomotor circuit is more complex with recurrently connected glutamatergic interneurons. In addition, interposed inhibitory interneurons that are part of the spinal locomotor circuit are not included in our model. Rhythmic activities generated by the spinal cord or computational models are often glutamate-dependent (Dale and Roberts, 1984; Buchanan and Grillner, 1987; Talpalar and Kiehn, 2010; Masino et al., 2012) with persistent sodium currents (Del Negro et al., 2002; Tazerart et al., 2008; Brocard et al., 2013; Shevtsova et al., 2020; Song et al., 2020; Verneuil et al., 2020) and electrical coupling (Hinckley et al., 2005; Wilson et al., 2005; Hinckley and Ziskind-Conhaim, 2006; Dougherty et al., 2013; Ha and Dougherty, 2018; Shevtsova et al., 2020) being particularly important for sustaining rhythmic activity. While our model included a persistent sodium current to support intrinsic bursting, the cell types, in addition to synaptic and intrinsic mechanisms that *generate* episodic bursting, remain to be systematically investigated. That said, similar theoretical modeling approaches have been implemented, and reduced HCO models of locomotion similar to ours mimic the behavior of more complex population-based models (Ausborn et al., 2018). Further, it is likely that other conductances contribute to the generation and modulation of episodic bursting given the degeneracies in mechanisms for rhythm generation in the STG (Prinz et al., 2004). Consistent with our HCO model, dopamine-evoked episodes are produced with inhibitory conductances that are maintained. However, synchronous rhythms with burst cycles consistent to those elicited by dopamine can also be elicited through blockade of inhibitory synaptic transmission. The intrinsic mechanisms of disinhibited bursting has been extensively explored (Ballerini et al., 1997; Rozzo et al., 2002; Taccola and Nistri, 2007; Kudela et al., 2009), with calcium-activated potassium (SK_*Ca*_) channels playing a key role in the control of both disinhibited (Mahrous and Elbasiouny, 2017) and episodic bursting (Mahrous and Elbasiouny, 2018). While our model does not include SK_*Ca*_ conductances, and given that dopamine reduces SK_*Ca*_-conductances in motoneurons (Han et al., 2007), exploration and integration of roles for SK_*Ca*_ channels in the generation and modulation of dopamine-evoked episodic bursting could serve as an interesting direction for future studies.

### Future Directions

Episodic swimming in larval zebrafish is generated by a distributed population of glutamatergic spinal neurons (Wiggin et al., 2014; Wahlstrom-Helgren et al., 2019) and episodic rhythms elicited in isolated rat spinal cords are diminished by blockers of glutamatergic transmission (Marchetti and Nistri, 2001). In support of those findings, our previous work demonstrated a role for NMDA receptors in maintaining episodic rhythms and enabling transitions to continuous rhythms (Sharples and Whelan, 2017). Future work adapting our model to include mutually excitatory synapses will shed light on the underlying mechanisms that contribute to episode initiation. Furthermore, complementary experimental approaches harnessing genetic tools available to study mice and zebrafish will also offer insight into putative “episode-generating” spinal interneurons. V3 interneurons are one population of interest, given their intrinsic and synaptic properties, and their role in supporting locomotor bouts (Zhang et al., 2008; Wiggin et al., 2012; Chopek et al., 2018; Danner et al., 2019). Alternatively, episodic bursting may be supported by astrocytes that have broad, distributed connectivity, regulate spinal motor circuits (Carlsen and Perrier, 2014; Acton et al., 2018; Broadhead and Miles, 2020; Carlsen et al., 2021), have well-established roles in regulating glutamatergic transmission at tripartite synapses (Perea et al., 2009), and form complex synaptic structures within the newborn and adult mouse spinal cord (Broadhead et al., 2020).

### Conclusion

Our modeling and *in vitro* experiments reveal that a single CPG could produce episodic activity and intra-episode bursting, and that degenerate mechanisms lead to transitions between silence, episodic, and continuous bursting through modulation of *I*_Pump_ or *I*_*h*_. While episodic and continuous patterns have been well documented in isolated developing mammalian spinal circuits (Barbieri and Nistri, 2001; Marchetti and Nistri, 2001; Gozal et al., 2014; Sharples and Whelan, 2017), these activities may not be restricted to developing nervous systems; there are examples of these different patterns occurring in adult fish, rodents, and cats (Müller et al., 2000; Crawley, 2007; Gabriel et al., 2011; Wiltschko et al., 2015; Mahrous and Elbasiouny, 2018). Our multidisciplinary work presents the first computational model capable of producing episodic bursting, which we validate with experimental recordings from isolated spinal cords. This study provides insight into the network mechanisms that govern the generation of context-dependent locomotor patterns produced during different states in freely behaving animals and in developing motor networks.

## Materials and Methods

### Ethical Approval and Animals

Experiments were performed on neonatal C57BL/6 (*n* = 112) mice, postnatal 0–4 days old (P0–P4). The University of Calgary Health Sciences Animal Care Committee approved all procedures (protocol number AC16-0182).

### Tissue Preparation

We anesthetized the animals via hypothermia by placing them at −20°C for 5–10 min. Animals were then decapitated, eviscerated, and the spinal column was pinned ventral side up in a dissecting dish lined with silicone elastomer (Sylgard, MilliporeSigma, Oakville, ON, Canada) and superfused with room temperature (21°C) carbogenated (95% O_2_–5% CO_2_) artificial cerebrospinal fluid (aCSF; in mM, 128 NaCl, 4 KCl, 1 MgSO_4_, 1.5 CaCl_2_, 0.5 Na_2_HPO_4_, 21 NaHCO_3_, 30 D-glucose; total osmolarity: 310–315 mOsm). The spinal cord was isolated by performing a ventral laminectomy. The nerve roots connecting the spinal cord to the vertebral column were subsequently cut, isolating the spinal cord. We then transferred the isolated spinal cord to a recording chamber perfused with carbogenated aCSF and placed it ventral side up. We gradually increased the bath temperature to 27°C (Whelan et al., 2000). The advantage of this method is that it is close to physiological temperature and avoids fluctuations in room temperature. We left the spinal cords to stabilize for 1 h before performing experiments.

### Electrophysiology

Extracellular electrophysiological recordings were obtained from ventral roots with tight-fitting suction electrodes fashioned from polyethylene tubing (PE50). Total signal amplification was 1,000×, including a pre-amplifier (10×) and second stage amplifiers (Cornerstone EX4-400 Quad Differential Amplifier) at 100×. Amplified signals were acquired in DC and digitized at 2.5 KHz (Digidata 1440A/1550B; Molecular Devices; Sunnyvale, CA, United States). We saved data acquired using Clampex 10.7 software (Molecular Devices) to a desktop computer for offline analysis.

### Pharmacology

Episodic rhythmicity was evoked by bath application of dopamine hydrochloride (50 μM; Sigma-Aldrich). We blocked the *I*_Pump_ with ouabain (100 nM–1 μM; Tocris), and *I*_*h*_ – producing hyperpolarization-activated cyclic nucleotide (HCN) channels with ZD 7288 (30 – 50 μM; Tocris) or with ivabradine (1 nM – 100 μM; Sigma-Aldrich). *I*_Pump_ was potentiated by monensin [monensin sodium salt, 2 μM dissolved in ethanol (0.03%), Sigma-Aldrich, M5273]. All pharmacological agents were prepared following solubility guidelines specified by their respective vendors. We were careful to ensure that volumes of drugs prepared in dimethyl sulfoxide (DMSO) did not exceed 0.04% (vol/vol) concentration in working solutions.

### Computational Model

The model consists of two identical and mutually inhibitory neurons, assembled as an HCO. Each neuron represents the activity of a population (flexor or extensor) of controlling interneurons located on the same side. It is described by a single equipotential electrical compartment and a single intracellular compartment used to compute Na^+^ concentration. Each neuron is equipped with ionic currents modeled using Hodgkin–Huxley formalism: slowly inactivating persistent Na^+^ current (*I*_*NaP*_), low-threshold slowly inactivating Ca^2+^ current (*I*_*CaS*_), a fast Na^+^ current (*I*_*NaF*_), a delayed rectifier-like K^+^ current (*I*_*KDR*_), a fast transient K^+^ current (*I*_*KA*_), an inhibitory synaptic current (*I*_*Syn*_), and a hyperpolarization-activated inward (*h*-) current, *I*_*h*_. The fast K^+^ current, *I*_*KA*_, was implemented using experimental data from (Hayes et al., 2008). Its activation is fast and is implemented as instantaneous. The equations for *I*_*NaF*_ and *I*_*KDR*_ were obtained from earlier work (Rybak et al., 2006) and implemented along with *I*_*CaS*_, *I*_*Syn*_, and *I*_*NaP*_ as described (Bondy et al., 2016). Our descriptions of the h-currents and leak currents were adapted from our previous study (Kueh et al., 2016) and had corresponding reversal potentials for Na^+^ and K^+^ components. The two components, Na^+^ and K^+^, of *I*_*h*_ shared the same conductance and activation values, but conductance was scaled differently, with 3/7 and 4/7 for the Na^+^ and K^+^ components, respectively. For the leak current, the ratio of the Na^+^ component to the K^+^ component was determined using a reference leak reversal potential, *E*_*LRef*_. Concerning the parameter choice, we were instructed by our previously developed model producing continuous bursting (Olypher et al., 2006; Bondy et al., 2016; Parker et al., 2018, 2019).

The membrane potential of each neuron in the HCO is governed by the following current conservation equation:


$$\begin{array}{c} C\frac{dV_{m}}{dt}=-[I_{NaF}+I_{NaP}+I_{KDR}+I_{KA}+I_{h-Na}+I_{h-K}+I_{CaS}\\ +I_{L-Na}+I_{L-K}+I_{Pump}+I_{Syn}] \end{array}$$

where *C* is the membrane capacitance in nF, *V_m_* is the membrane potential in mV, t is time in seconds, and *I*_*xyz*_ are ionic membrane currents: They are described by the following formula:


INaF=g¯NaFmhNaFNaF∞3[Vm-ENa],

$$I_{NaP}={\bar{g}}_{NaP}m_{NaP}h_{NaP}\left[V_{m}-E_{Na}\right],$$

$$I_{KDR}={\bar{g}}_{KDR}m_{KDR}^{4}\left[V_{m}-E_{K}\right],$$

$$I_{KA}={\bar{g}}_{KA}m_{KA\infty }h_{KA}\left[V_{m}-E_{K}\right],$$

$$I_{h-Na}=\frac{3}{7}{\bar{g}}_{h}m_{h}^{2}\left[V_{m}-E_{Na}\right],$$

$$I_{h-K}=\frac{4}{7}{\bar{g}}_{h}m_{h}^{2}\left[V_{m}-E_{K}\right],$$

$$I_{CaS}={\bar{g}}_{CaS}m_{CaS}^{3}h_{CaS}\left[V_{m}-E_{Ca}\right]$$

and two components of the leak current:


$$I_{L-Na}=g_{L-Na}\left[V_{m}-E_{Na}\right]$$

$$I_{L-K}=g_{L-K}\left[V_{m}-E_{K}\right]$$

where leak conductances were computed with the reference values of equivalent total leak conductance *g_L_* and reversal potential *E*_*LRef*_ using the Na^+^ reversal potential:


$$E_{NaRef}:g_{L-Na}=g_{L}\left[\frac{E_{LRef}-E_{K}}{E_{NaRef}-E_{K}}\right]\mathrm{and}$$

$$g_{L-Na}=g_{L}\left[\frac{E_{LRef}-E_{NaRef}}{E_{K}-E_{NaRef}}\right].$$

The equation for the pump current was adapted from Cressman et al. (2009); it has maximal value *I*_*PumpMax*_ = 40.26 pA and is activated by [Na^+^]_*i*_ and [K^+^]_*e*_ with concentrations of half-activation 25 and 6 mM, respectively:


IPump=[IPumpMax1+exp(25-[Na]+i3)][11+exp(6-[K]+e)]

*I*_*Syn*_ is the synaptic current:


$$I_{Syn}={\bar{g}}_{Syn}m_{SynPre}\left[V_{m}-E_{Syn}\right]$$

where *m*_*SynPre*_ is a synaptic activation variable. It is governed by the membrane potential of the presynaptic cell and is correspondingly described as a state variable of the presynaptic cell. The gating variables are mostly governed by equations using the following Boltzmann function: $f_{\infty }\left(a,V_{\frac{1}{2}},V_{m}\right)=\frac{1}{1+exp\left(\frac{V_{m}-V_{\frac{1}{2}}}{a}\right)}$


$$\frac{dh_{NaF}}{dt}=\left[f_{\infty }\left(7,26,V_{m}\right)-h_{NaF}\right]/\tau_{h_{NaF}}$$

$$\frac{dm_{NaP}}{dt}=\left[f_{\infty }\left(-4.1,-43,V_{m}\right)-m_{NaP}\right]/0.001$$

$$\frac{dh_{NaP}}{dt}=\left[f_{\infty }\left(5,-57,V_{m}\right)-h_{NaP}\right]/0.1$$

$$\frac{dm_{KDR}}{dt}=\left[f_{\infty }\left(-15,-18,V_{m}\right)-m_{KDR}\right]/\tau_{m_{KDR}}$$

$$\frac{dh_{KA}}{dt}=\left[f_{\infty }\left(5,-59.8,V_{m}\right)-h_{KA}\right]/0.02$$

$$\frac{dm_{CaS}}{dt}=\left[f_{\infty }\left(-4.27,-45.6,V_{m}\right)-m_{CaS}\right]/\tau_{m_{CaS}}$$

$$\frac{dh_{CaS}}{dt}=\left[f_{\infty }\left(0.86,-56.34,V_{m}\right)-h_{CaS}\right]/0.34$$

$$\frac{dm_{h}}{dt}=\left[\frac{1}{1+2exp\left(221.4\left[V_{m}+52.1\right]\right)+exp\left(615\left[V_{m}+52.1\right]\right)}-m_{h}\right]/\tau_{h}$$

$$\frac{dm_{Syn}}{dt}=\left[f_{\infty }\left(-0.4,-25,V_{m}\right)-m_{Syn}\right]/0.05$$

$$m_{NaF\infty }=f_{\infty }\left(-7.8,-15,V_{m}\right),m_{KA\infty }=f_{\infty }\left(-10,-20,V_{m}\right),$$

$$\tau_{h_{NaF}}=\frac{0.03}{exp\left(\frac{V_{m}+43}{15}\right)+exp\left(\frac{V_{m}+43}{-16}\right)}$$

$$\tau_{m_{KDR}}=\frac{0.007}{exp\left(\frac{V_{m}+43}{40}\right)+exp\left(\frac{V_{m}+43}{50}\right)}$$

$$\tau_{m_{CaS}}=\frac{0.001}{\frac{0.02\left[V_{m}+48.01\right]}{1+exp\left(\frac{V_{m}+48.01}{4.5}\right)}+\frac{0.05\left[V_{m}+51.01\right]}{1+exp\left(\frac{V_{m}+51.01}{4.5}\right)}}$$

$$\tau_{h}=0.5+\frac{1}{1+exp\left(100\left[V_{m}+73\right]\right)}$$

Intracellular potassium concentrations ([K^+^]_*i*_) and extracellular potassium concentration ([K^+^]_*e*_) are fixed parameters. Our model has a dynamic intracellular Na^+^ concentration ([Na^+^]_*i*_) and a fixed extracellular Na^+^ concentration ([Na^+^]_*e*_). To compute [Na^+^]_*i*_, the model accounts for Na^+^ influx carried by the Na^+^ currents (*I*_*NaP*_ and *I*_*NaF*_) and Na^+^ components of leak (*I*_*L–Na*_) and h- (*I*_*h–Na*_) currents, and outflux produced by the Na^+^/K^+^ ATPase pump current (*I*_Pump_):


d[Na]+id⁢t=M[[Na]+e-[Na]+i]-α[Ih-N⁢a+IL-N⁢a+IN⁢a⁢F+IN⁢a⁢P3IP⁢u⁢m⁢p]

where $\alpha =\frac{1}{vF}=4.682255338155292$ mM/pA/s, v is intracellular volume and F is Faraday’s constant. We simulate the application of monensin with the term that describes Na^+^ diffusion proportional to a parameter of the monensin rate constant (M). This rate constant is *M* = 0 1/s unless monensin is applied.

Reversal potentials for Na^+^ and K^+^ currents are computed as the Nernst potentials $E_{Na}=\frac{RT}{F}ln\frac{{\left[Na\right]}_{e}}{{\left[Na\right]}_{i}}$ and $E_{K}=\frac{RT}{F}ln\frac{{\left[K\right]}_{e}}{{\left[K\right]}_{i}}$ with $\frac{RT}{F}=26.45mV$, using fixed [Na]+e=120mM, computed state variable [Na]+i, and fixed [K]+e=9mM and [K]+i=130mM. Canonical values of other model parameters are *C* = 0.001*nF*, ${\bar{g}}_{NaF}=105nS,$${\bar{g}}_{NaP}=$4.97 nS, ${\bar{g}}_{KDR}=79nS$, ${\bar{g}}_{KA}=1.13nS,$${\bar{g}}_{h}=0.34nS,$${\bar{g}}_{CaS}=3.3nS,$*E*_*Ca*_=160*mV*, *g*_*L*_ = 1.88*nS*, *E*_*LRef*_ = −55*mV*, *E*_*NaRef*_ = 65*mV*, ${\bar{g}}_{Syn}=1.02nS,$*E*_*Syn*_ = −70*mV*.

We analyzed a single, decoupled cell in the model as well, with all of the same canonical parameters, but we applied a scaling factor to the time constant of *m*_*h*_, so that the equation for *m*_*h*_ became the following.


$$\begin{array}{c} \frac{dm_{h}}{dt}=\left[\frac{1}{1+2exp\left(221.4\left[V_{m}+52.1\right]\right)+exp\left(615\left[V_{m}+52.1\right]\right)}-m_{h}\right]/\\ \left({\left(scalefactor\right)}^{*}\tau_{h}\right) \end{array}$$

We increased the scale factor from 1 to 50 to investigate the effects of increasing τ_*h*_ in the single cell model. Additionally, we built a reduced version of the single cell model where *m*_*h*_ was held constant and varied as a parameter. We referred to this model as the reduced constant-*m*_*h*_ model. We compared the manual variation of *m*_*h*_ in this reduced model to the full single cell model with a scale factor of 1 and with a scale factor of 50.

### Data Analysis

The model was implemented in the C programming language. Integration was performed using the explicit embedded Runge–Kutta Prince–Dormand (8,9) method with an absolute error tolerance of 10^–8^, relative error tolerance of 10^–9^, and an initial step size of 10^–8^ (the GNU scientific library; Galassi et al., 2002). The model was integrated at each parameter value investigated for 5,000 s before analyzing to ensure that the model had reached its stable state. Obtained model data were analyzed using custom-written MATLAB scripts (Mathworks Inc., Natick, MA, United States).

Episodes of rhythmicity were analyzed using custom-written MATLAB scripts to detect episode onset and offset. In experimental data, we also measured amplitude. We determined episode onset and offset from DC signals that were detrended, band-pass filtered (0.01–1 Hz), and smoothed with a Gaussian-weighted moving average. We then calculated EP, EP-CV, ED, and IEI in Excel from episode onset and offset times. Briefly, EP was defined by the onset of one episode to the next, ED was defined as the onset and offset of each episode, and the interepisode interval defined as the period of non-bursting activity between from the offset of one episode and the onset of the following episode. The EP coefficient of variation (EP-CV) was set to distinguish regular and irregular patterns of episodic bursting. Parameters are reported in text as mean ± SD. Similar analysis was done for modeling data using HCO membrane potentials to detect bursts and episodes of bursting activity.

### Statistical Analysis

We analyzed changes in characteristics of episodic rhythmicity recorded in ventral roots over time using one-way or two-way repeated measures analyses of variance (ANOVA), as appropriate. Pharmacological manipulations of spinal circuits were compared to appropriate time-matched preparations using unpaired *t*-tests. Data that violated assumptions of normality (Shapiro–Wilk test) or equal variance (Brown–Forsythe test) were analyzed via nonparametric Mann–Whitney *U* (if two groups) or Kruskal–Wallis (if more than two groups) tests. All effects surpassing a significance threshold of *p* < 0.05 were further examined with Holm–Sidak *post hoc* tests to compare all treatment conditions to the appropriate normalized time-matched vehicle control.

## Data Availability Statement

The models are described in the article. Their implementations in C are available on a model repository at http://modeldb.yale.edu/267253.

## Ethics Statement

The animal study was reviewed and approved by University of Calgary, Health Sciences Animal Care Committee.

## Author Contributions

SS, JM-C, AL, NC, and AS performed *in vitro* experiments. SS and JM-C analyzed the data with code written by LY. AV, JP, and GC developed and analyzed the computational model. SS, JP, AV, JM-C, GC, and PW prepared figures and wrote the manuscript. All authors approved the final version of the manuscript. SS, GC, and PW conceived and designed the research.

## Conflict of Interest

The authors declare that the research was conducted in the absence of any commercial or financial relationships that could be construed as a potential conflict of interest.

## Publisher’s Note

All claims expressed in this article are solely those of the authors and do not necessarily represent those of their affiliated organizations, or those of the publisher, the editors and the reviewers. Any product that may be evaluated in this article, or claim that may be made by its manufacturer, is not guaranteed or endorsed by the publisher.
