# Supplementary material for: Contributions of h- and Na+/K+ Pump Currents to the Generation of Episodic and Continuous Rhythmic Activities
Source: Front Cell Neurosci. 2022 Feb 4;15:715427. doi: 10.3389/fncel.2021.715427 (PMC8855656; doi:10.3389/fncel.2021.715427)
Supplement: Supplementary file 1 [file Data_Sheet_1.pdf]

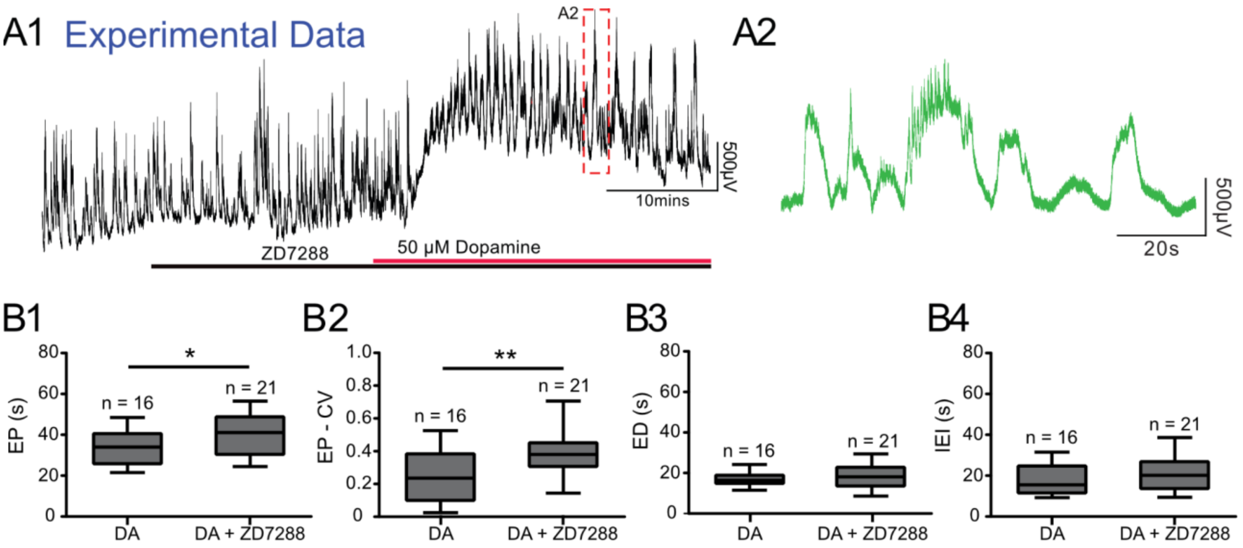


**Supplemental Figure 1:** ZD7288 produces a disrupted pattern resembling spontaneous activity. (A) Extracellular neurograms (A1) recorded from a single ventral root show episodic rhythmicity evoked by dopamine (black bar) in the presence of the HCN channel blocker ZD7288 (30-50 µM, red bar). A2 expands the inset in A1. B. ZD7288 increased the episode period (EP; B1) and episode period variability (EP-CV; B2), but did not alter episode duration (ED; B3), or interepisode interval (IEI; B4). Box and whisker plots depict interquartile range (boxes), median (horizontal black lines), maximum, and minimum values in the data range (whiskers) for episodic rhythms evoked by dopamine alone (n = 16), or in the presence of ZD7288 (n = 21) in separate sets of experiments; asterisks indicate significance of unpaired t-tests (* $p < 0.05$; ** $p < 0.01$).
